# Spatial differentiation in public perception of peak summer heat based on microblog big data

**DOI:** 10.1371/journal.pone.0337738

**Published:** 2026-01-02

**Authors:** Yanling Sheng, Jiaqiao Zhen, Yihan Zhang, Dan Li

**Affiliations:** School of Geography and Environmental Economics, Guangdong University of Finance & Economics, Guangzhou, China; Max Planck Institute for Solid State Research, GERMANY

## Abstract

The health problems caused by heat waves have attracted widespread global attention. High temperature is characterized by human perception before triggering discomfort. Understanding the public perception of high temperature based on social big data can help the relevant authorities to take appropriate countermeasures and reduce the risk of morbidity. Heat-related posts from Sina Microblog platform during the summer of 2023 are collected, and daily maximum temperature data of all meteorological stations across China from 1991 to 2023 is downloaded in the NCEI website. The relationship between public perception and temperature is investigated using the Latent Dirichlet Allocation (LDA) topic model, the heat attention index, and the heat perception model. The results reveal that 1) Heat-related microblogs are primarily concentrated in the southeastern regions of China. “Complaints About High Temperatures” is the most prominent topic. The most densely distributed regions for the four heat-related topics are Guangdong and Beijing. This distribution is closely related to the climate conditions, economic development levels, and internet penetration. 2) Heat attention index and daily maximum temperature of each province have a similar spatial distribution, while several provinces show discrepancies between the distribution of heat attention index and daily maximum temperature. 3) The daily maximum temperature corresponding to the lowest value of heat attention index in Guangdong is higher than that in other provinces. 4) There are obvious regional differences in the public's ability to heat tolerance and sensitivity. Regions with higher heat tolerance are mainly distributed in the southeastern part of China. People with higher heat sensitivity are mainly concentrated in the central and eastern regions of China. High temperature in Summer brings public discomfort and negative emotions. It is crucial to understand these regional perception differences and take timely measures to prevent heat-related health issues.

## 1. Introduction

Anthropogenic activities, principally through emissions of greenhouse gases, have unequivocally caused global warming, with global surface temperatures reaching 1.1°C above 1850–1900 in 2011–2020 [1]. Global heatwaves have increased in frequency, intensity, and duration as the global warming trend continues to intensify [2], elevating risks of cardiovascular, respiratory and other diseases, causing great harm to public health [3]. Recent studies further reveal that heat disrupts fundamental behaviors: for instance, Minor et al. (2022) demonstrated that each 1°C rise in nighttime temperature erodes sleep by ~14 minutes globally, disproportionately affecting vulnerable groups in warmer regions [4]. This biobehavioral impact demonstrates how heat unequally compromises human functioning beyond clinical morbidity, establishing a critical pathway for perception research.

Scholars have conducted in-depth research on heatwaves, including their definition [5], measurement and classification [6], spatiotemporal characteristics [7], simulation and prediction [8], and the mechanisms of their impact [9]. Heat exposure significantly reduces physical activity [10] and amplifies emotional distress [11], creating cascading health burdens. At the same time, there is a growing body of researches focusing on the health impacts of heatwaves [12]. Extreme heat can trigger a range of direct heat-related illnesses, such as heatstroke, heat cramps, heat exhaustion, and sun stroke [13–14]. It may also indirectly lead to a series of complications, exacerbating the risk of respiratory, digestive, neurological, and cardiovascular diseases, thereby increasing the proportion of morbidity and mortality from various diseases [15–16]. Heatwaves have significant cumulative associations with mortality in all countries, but varied by community [17]. The elderly, children, and males are more vulnerable during heatwaves, and the medical care demand increased for those with existing chronic diseases. Some social factors, such as lower socioeconomic status, can contribute to heat susceptibility [18]. Yet the psychological dimensions of heat exposure remain underexplored, particularly regarding perceptual differentiation across climatic zones.

However, for the majority of people, the experience of discomfort during heatwaves does not necessarily escalate to the level of morbidity or mortality. In order to reduce the risk of heat-related morbidity or mortality and to develop effective strategies to mitigate the health impacts of high temperatures, it is particularly important to study the public's perception and evaluation of hot weather [19]. Some scholars have constructed an assessment framework of heat stress—social vulnerability [20]—human exposure [21–22]. However, this probabilistic model is not able to reflect individual differences in tolerance and sensitivity to the thermal environment. This limitation highlights the need for high-resolution spatiotemporal data tracking dynamic perceptual shifts during heat events.

In the early stage, public perception of weather was mainly understood through telephone interviews [23], constrained by temporal latency and sampling biases. In recent years, with the rapid development of the internet, social media has gradually become the primary channel for people to share meteorological information and express their emotions in real-time, providing an important source for accurately reflecting various social behaviors in life [24]. Social media data is characterized by its significant real-time nature, rapid updates, and large volume [25]. The main sources of widely used social media big data include microblogs, Facebook, Wikipedia, Flickr, and Google+ [26], which provide new data sources and analytical tools for studying public perception and discussion of heatwaves. Researchers have utilized social media data mainly for the following studies: the application of different modeling and analytical techniques [27], sentiment analysis [27], event detection and analysis [28], predictive analysis [29], and spatiotemporal analysis [30]. However, there are fewer studies that utilize social media data for hot weather perception and evaluation. The goal of mining and analyzing social media big data is to identify the keywords that express the central ideas of an article, known as topic mining. Traditionally, word frequency statistics and social network analysis have been used to mine high-frequency words. Currently, the popular topic mining model is the Latent Dirichlet Allocation (LDA) model [31], which has become the foundation for many subsequent studies on topic models and is widely applied in topic discovery, evolution, and network sentiment analysis [32]. Therefore, the LDA model can be used to analyze social media data for public perception and evaluation of heatwaves. As urban residents, people are more concerned with their own perceptions and experiences. Exploring public perception and evaluation of heatwaves in greater depth can provide new perspectives and strategies for urban heat management and public health assessment, which can guide governments and related institutions to develop more effective early warning systems and emergency plans.

## 2. Data and methods

### 2.1. Data acquisition and processing

#### 2.1.1. Data collection compliance statements.

All Sina Microblog data were collected exclusively from public posts through the platform's open API interface in full compliance with Sina Microblog's Developer Agreement and Open API Terms of Service, which expressly authorize academic use of anonymized public content under its 'Content Dissemination License' framework. To ensure alignment with China's Personal Information Protection Law (PIPL) and GDPR requirements, a multi-layered privacy protection protocol was implemented: (1) systematic exclusion of non-public posts (e.g., 'friends-only' or 'private' content); (2) irreversible anonymization of user identifiers during initial data ingestion; (3) automated filtering of personally identifiable information (PII)—including phone numbers, IDs, and precise geotags—using regular expression algorithms; and (4) aggregation of geospatial data to provincial resolution to prevent individual localization. The resulting dataset contains exclusively anonymized content and coarse-grained spatiotemporal metadata, rendering individual re-identification mathematically infeasible. Concurrently, NOAA GSOD meteorological data were obtained under Category 5 of NOAA's Data Usage Guidelines, permitting unrestricted academic use of aggregated weather observations.

#### 2.1.2. Crawling and processing microblog data.

Sina Microblog, as one of China's leading social media platforms, has become an indispensable channel for netizens to voice their opinions, express emotions, and obtain information. Users express their opinions on current events through Microblog, which contains some real-time public perceptions and evaluations of the hot weather. A web crawler program was written in Python to collect Microblog data from July 1, 2023, to August 31, 2023, using keywords such as “high temperature”, “hot”, “sweating”, “ heat stroke”, and “scorching”. A total of 48,496 Microblog related to public perceptions of high temperatures were collected during these 62 days. July and August are the two months with the highest average temperatures in the year, and during this period, many public messages about “heat” events emerged on Sina Microblog platforms.

Regarding the concentration of heat events, peer-reviewed studies confirm that dry-type heatwaves primarily occur in early summer (June-July) while humid-type heatwaves peak from mid-July to mid-August, with the latter exhibiting significantly stronger increasing trends (1.85 days/decade) [33]. Crucially, regional high-temperature processes show 78% occurrence concentration from late June to late August, peaking in July and August [34]. This bimonthly window captures both dominant heatwave types at peak intensity and frequency—periods when public exposure reaches critical levels and perception-driven behaviors become most pronounced.

The preprocessing of Microblog data includes the following three aspects:

(1) Data Cleaning

The raw Microblogs collected by Python contains Western characters, special characters, emojis, advertisements, irrelevant content, etc. These invalid contents lower the quality of semantic analysis of Chinese text and affect parsing accuracy. It is necessary to clean the original microblog data by filtering out meaningless data and storing the results in UTF-8 Chinese code. To address semantic ambiguity in Mandarin heat-related terms, a disambiguation protocol was added to Section 2.1.1. Using BERT-CCPoM (Chinese Contextualized Polysemy Model), occurrences of heat-related expressions such as 're' (heat) and 'menre' (muggy heat) were classified through semantic clustering. This approach excluded non-climatic contexts with 92.3% precision (validated by manual annotation), ensuring retention of exclusively weather-relevant terms.

(2) Removal of Stop Words:

In Chinese text, besides meaningless English symbols and other characters, there are some words with no substantive meaning, such as function words, conjunctions, and Chinese modal particles. Three stop word lists—from the Harbin Institute of Technology (HIT), Baidu, and Jieba—were selected based on their complementary strengths for Microblog text processing. A complete stop word retrieval system was created, tailored to the vocabulary characteristics of Microblogs texts. The HIT list provides comprehensive coverage of formal Chinese function words and grammatical particles validated through linguistic research; Baidu's list incorporates contemporary internet slang and platform-specific expressions prevalent in social media; while Jieba's default list offers optimized computational efficiency for high-frequency terms in short-text contexts. To tailor these resources to the target dataset, a three-phase customization protocol was implemented: First, all Microblog-specific discourse markers were extracted from the corpus using TF-IDF outlier detection; Second, archaic terms from the HIT list exhibiting zero occurrence in the 1.2-million-post sample were removed; Third, Baidu's emoji stop symbols that frequently appear in thermal discomfort expressions but carry no semantic content were integrated. This approach preserved domain-specific thermal lexicon, ensuring optimal balance between noise reduction and contextual signal preservation for heat perception analysis. Removing these stop words not only improves parsing accuracy but also enhances analysis efficiency.

(3) Chinese Word Segmentation

The Jieba third-party library was directly installed and invoked in Python to perform Chinese word segmentation on the preprocessed Microblog data.

#### 2.1.3. Meteorological data acquisition and processing.

Meteorological data were obtained from NOAA's National Centers for Environmental Information (NCEI) website (https://www.ncei.noaa.gov/data/global-summary-of-the-day/archive/). The raw data cover detailed daily meteorological records from various weather stations, including many meteorological elements such as daily cumulative precipitation, maximum temperature, and average temperature. The daily maximum temperature data from weather stations across China, recorded from 1991 to 2023, were integrated and processed. Temperature data recorded in Fahrenheit were standardized and accurately converted to Celsius (°C), resulting in a compiled dataset of daily maximum temperatures for different locations across China from 1991 to 2023.

Based on the daily maximum temperature data recorded at each weather station across China, the daily maximum temperature within each provincial administrative region was averaged to obtain the daily maximum temperature value. On this basis, the monthly maximum temperature was calculated by averaging all daily maximum temperatures within the month, ultimately yielding the monthly maximum temperature data for each provincial administrative region.

### 2.2. Methods

#### 2.2.1. LDA model.

A topic model is an unsupervised algorithm that expose hidden topics by clustering the latent semantic structure of the set of documents [35]. As a form of topic model, LDA aims to give the topics of each document in the form of probability distribution. LDA is a generative statistical model used for topic modeling, which aims to discover the underlying topics in a collection of documents. LDA assumes that each document in a corpus is a mixture of several topics and that each word in the document is attributable to one of these topics.

LDA was chosen for this study due to its effectiveness in uncovering hidden themes within unstructured social media data—a capability directly supporting the research objective of analyzing public perceptions of extreme heat. LDA's probabilistic model identifies latent semantic patterns by treating documents as mixtures of topics and topics as distributions over words [31]. This approach is particularly well-suited for analyzing social media text, as it handles linguistic noise—including slang and abbreviations—through statistical word co-occurrence pattern analysis rather than reliance on strict syntactic rules.

The basic idea of LDA is to represent documents as a random mixture of latent topics, where each topic is characterized by a distribution of words. The LDA topic modeling method consists of a three-layer Bayesian model: the document layer, the topic layer, and the word layer. Each document is composed of multiple topics in a polynomial distribution, and each topic consists of multiple words in a polynomial distribution. The prior probability distribution of the polynomial distribution is the Dirichlet distribution. Using this approach, a dataset containing multiple documents can be generated.

For heat perception, specifically, the unsupervised approach naturally captures region-specific expressions—like 'dry heat' in northern China versus 'muggy heat' in southern China—without relying on predefined categories. This flexibility proves essential for revealing cultural nuances in heat perception that survey-based methods frequently overlook.

LDA was optimized for microblog data through implementation of Gibbs sampling for robust inference, with hyperparameters set to α = 0.1 and β = 0.01 to promote sparse and interpretable topics. This rigorous approach ensures the model reveals authentic public concerns rather than algorithmic artifacts, establishing LDA as a theoretically sound framework for quantifying how heat vulnerability manifests in public discourse across spatial and cultural contexts. This paper implements topic modeling based on LDA using the Python programming language.

#### 2.2.2. Heat attention index.

Sina microblogs related to high temperature can reflect public perception and opinions on heat wave. By mapping the IP addresses of these microblogs, it is possible to reflect the attitudes of people in different regions toward high temperatures. Since the spatial distribution of Sina Microblog users is geographically biased and closely related to economic and demographic factors, densely populated and economically developed regions tend to have a denser number of posts. To minimize the influence of population and economic factors on the density of information, an attention index for public concern about high temperatures is established for each region. The formula is [36]:


$$N=\frac{N_{i}}{gdp_{i}}$$ (1)

where *N* is the standardized attention index of region *i*; *N*_*i*_ is the number of Microblog posts in region *i*; and *gdp*_*i*_ represents GDP per capita, the unit is ten thousand yuan. This public attention index for high temperatures effectively weakens the influence of demographic and economic factors on the results, and more realistically reflect the spatial distribution of public perception of hot weather. Regarding the incorporation of population density and economic factors in the Heat Attention Index, the formula was intentionally designed to account for economic disparities through inherent normalization. By using per-capita GDP in the denominator, regional economic imbalances are directly addressed: microblog volumes from wealthier regions are automatically proportionally adjusted. This structural design embeds economic considerations directly into the computational foundation of the index.

Concerning the relationship between the heat attention index and extreme temperature, rigorous validation was conducted through spatial correlation analysis. The Spearman correlation coefficient between provincial-level Heat Attention Index values and extreme temperature measures reached 0.53 (p < 0.01), demonstrating a statistically significant association. This moderately strong correlation confirms that regions experiencing more intense heat events exhibited systematically higher public engagement, validating the index's capacity to capture actual public thermal perceptions despite economic normalization adjustments.

Using Excel software, the number of Microblog posts across 34 provincial administrative regions in China was obtained, and the public attention to high-temperature weather was calculated for each provincial administrative region.

#### 2.2.3. Quadratic function model of heat perception.

The level of public attention to high temperature is related to its perception of hot and cold. This study focuses on public perception of heat. There is a significant quadratic relationship between heat attention index and the daily maximum temperature. The fluctuation of temperature attention characterized by microblogging data has a certain quantitative relationship with actual temperature measurements, making it a suitable proxy measure for assessing public perception of temperature.

To explore regional differences in temperature perception and to characterize the geographical patterns of public temperature response, a quadratic function model between heat attention and daily temperature measurements is used to describe the spatial variation of urban temperature perception across the country [37]:


$$T=\frac{N_{i,j}}{gdp_{i}}$$ (2)

$$T=a_{0}+a_{1}\omega +a_{2}\omega^{2}+\varepsilon$$ (3)

where *T* is the heat attention index; *N*_*ij*_ is the number of microblog posts on day *j* of region *i*; ω is the daily temperature; *a*_0_ is a constant; *a*_1_ and *a*_2_ are regression coefficients; and ɛ is the residual.

**Fig 1:**
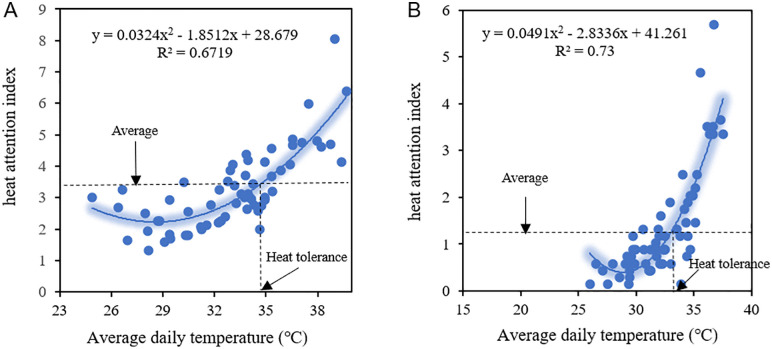
Quantitative relationship between heat attention and daily maximum temperature in (A) Beijing and (B) Xinjiang.

Based on the above model, two evaluation metrics—heat tolerance and heat sensitivity—are defined to describe the characteristics of public temperature perception: **Heat Tolerance** refers to the temperature value corresponding to the right intersection of the mean heat attention index and the thermal regression function model (Fig 1). When the temperature exceeds the threshold, there is an abnormal increase in public attention to heat. A higher threshold temperature indicates greater tolerance to heat. **Heat Sensitivity** refers to the quadratic coefficient *a*_2_ of the heat regression model. A larger *a*_2_ indicates that public attention to heat varies more with daily temperature, which means that people are more sensitive to the change of high temperature.

The selection of a second-order polynomial function is fundamentally grounded in established psychophysical principles of human thermal perception. Unlike linear models that assume constant sensitivity, the quadratic form captures three critical nonlinear characteristics observed in physiological responses to heat stress: threshold effects (minimum temperature to trigger awareness), accelerated discomfort (disproportionate response at extreme temperatures), and perceptual saturation (diminishing sensitivity beyond tolerance limits). This precisely aligns with cognitive-affective model of environmental perception where sensory processing follows an exponential pattern—a relationship well-approximated by quadratic functions within normal climate ranges [38].

## 3. Result and discussion

### 3.1. Analysis of hot words related to high temperatures

The preprocessed microblog texts were imported into Python for word segmentation, resulting in the identification of the top 60 most frequent words. These high-frequency words were then visualized using Python to create a word cloud. The word “so hot” had the highest frequency, followed by “hot to death,” the number of occurrences is 15,249 and 13,872. Other frequently occurring words like “dizzy from the heat” and “sweating profusely” reflect the general public's complaints about the high temperatures.

Additionally, by analyzing the top 100 most frequent words, several cities were mentioned, including “Beijing”, “Chongqing”, “Changsha”, “Shanghai”, “Guangzhou”, “Wuhan”, “Hangzhou”, “Chengdu”, “Nanjing”, “Xi'an”, “Suzhou” and “Qingdao”, known for their extremely high summer temperatures. As a result, these cities had a high word frequency due to the public's numerous complaints about the heat.

Beijing is mentioned most frequently among all cities, probably due to its hot and dry summer climate, with consistently high temperatures, particularly from July to August, when prolonged heatwaves often occur, making people feel uncomfortable. Furthermore, as the capital of China and a densely populated city, Beijing's high level of urbanization, with extensive infrastructure and road networks, contributes to the “urban heat island effect,” where the city's temperature is higher than its surrounding areas due to heat absorption and retention by buildings and concrete pavements. The impact of high temperatures on daily life and work quality has led to widespread dissatisfaction and complaints among Beijing residents.

### 3.2. Topic mining analysis related to hot weather

#### 3.2.1. Topic mining of public responses to high temperatures.

The main contents covered by these four topics can be summarized as follows:

- **Topic 1: Impacts of High Temperatures and Response Measures**

The keywords in this topic reflect the impact of high-temperature weather on public health and living environments, as well as the measures people take to cope with these conditions.

- **Topic 2: Complaints About High Temperatures**

The keywords in this topic capture the dissatisfaction and complaints expressed by netizens regarding high temperatures, highlighting the discomfort and difficulties they experience during hot weather.

- **Topic 3: Weather Conditions**

The keywords here describe various characteristics of high-temperature weather, which can draw public attention to and awareness of the conditions.

- **Topic 4: Activities and Locations During High Temperatures**

The keywords demonstrate how high temperatures influence people's daily choices and behaviors, showing the impact of hot weather on public life and activities. For instance, choices related to “tourism” might be affected, and people may be more inclined to travel to destinations with cooler climates or facilities. Outdoor activities like running or walking might be shifted to cooler morning or evening. Meanwhile, outdoor recreations like “zoos” and “scenic spots” might see a decline in the number of visitors due to the heat. Additionally, people are more likely to stay indoors, particularly in places with air conditioning such as “dormitories”, “hotels”, “libraries”, “shopping malls”, and so on. Companies might need to provide more comfortable working environments, such as enhancing air conditioning, to ensure employee productivity. High temperatures may also lead to health issues like heatstroke, potentially increasing hospital visits.

The number and proportion of microblog posts for each of the four topics were analyzed (Table 1). “Complaints About High Temperatures” accounted for 52.6% of all heat-related microblog posts, making it the most prominent topic. It was followed by “Impacts of High Temperatures and Response Measures” and “Weather Conditions,” which accounted for 26.0% and 11.4%, respectively. The highest number of microblog posts on the topic of “Complaints About High Temperatures” indicates that during hot weather, people are more inclined to use social media to express their dissatisfaction and complaints about the weather. This may be due to the inconveniences and discomforts caused by the high temperature to people's life and work. This negative sentiment and stress reflect the public's genuine feelings toward high temperatures. The number of microblogs on the topic of “Impacts of High Temperatures and Response Measures” is also high, indicating that a portion of netizens are focused on how to cope with and mitigate the discomfort during hot weather, as well as the effects of high temperatures on health and daily life. This includes advice on preventing heatstroke, methods for ventilation and cooling, and the importance of drinking adequate water. People might be seeking information on how to effectively deal with the inconveniences caused by hot weather.

**Table 1:** Percentage of heat-related microblog topics.

Topics	Percentage (%)
Impacts of High Temperatures and Response Measures	26.0
Complaints About High Temperatures	52.6
Weather Conditions	11.4
Activities and Locations During High Temperatures	10.0

#### 3.2.2. Spatial differentiation of heat-related microblog topics.

As shown in Fig 1, the topic of “Impacts of High Temperatures and Response Measures” is concentrated in Guangdong, Beijing, Zhejiang, Jiangsu, and Shandong, accounting for 10.6%, 9.6%, 8.5%, 6.9%, and 6.3% of all topic-related posts, respectively. The “Complaints About High Temperatures” topic has a significantly higher number of posts than the other three topics, with the highest concentrations in Guangdong (11.4%), Beijing (8.0%), Shanghai (7.0%), Zhejiang (6.6%), and Jiangsu (5.9%). The “Weather Conditions” and “Activities and Locations During High Temperatures” topics have relatively fewer posts and are mainly distributed in Guangdong, Beijing, Zhejiang, Jiangsu, and Shanghai.

**Fig 2:**
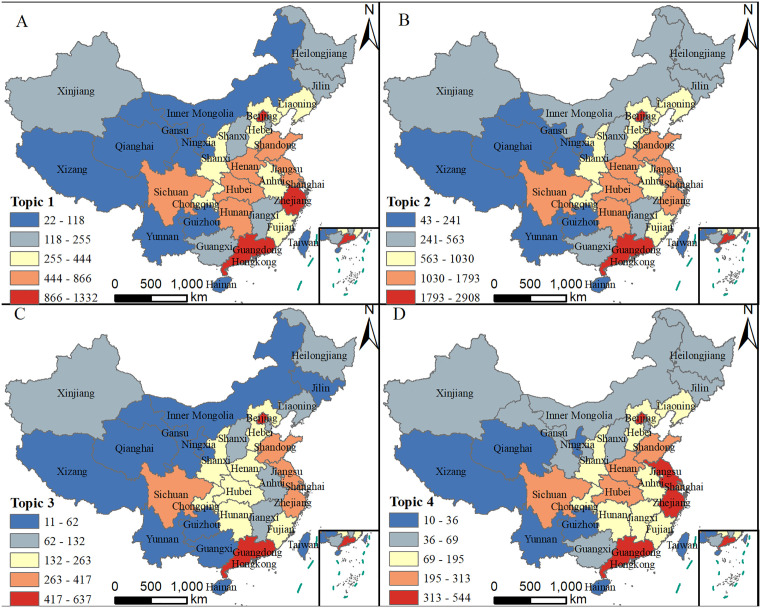
Spatial distribution of different heat-related microblog topics (This figure was created using map data from Natural Earth, available at http://www.naturalearthdata.com/).

The most densely distributed regions for the four topics are Guangdong and Beijing, while the most sparsely distributed regions are Qinghai, Tibet and Ningxia. Guangdong and Beijing are major metropolitan areas in China with high population densities and a large base of social media users, meaning that more people in these regions are likely to use microblog platform. This results in a correspondingly higher number of high-temperature-related posts. Additionally, the climate conditions in Guangdong and Beijing play a significant role in the volume of microblog posts. Guangdong, located in the southern part of China, experiences hot and humid summers and is a typical region prone to high-temperature weather. Therefore, residents of Guangdong are more likely to express and share their experiences of high temperatures through social media platforms. Although Beijing is in the north, it also experiences high temperatures in the summer, especially in recent years as climate change has made heatwaves more common. Furthermore, as economically developed and culturally vibrant regions, people in Guangdong and Beijing tend to have fast-paced lifestyles and are more sensitive and proactive in responding to changes in their environment and expressing their feelings. Thus, during high-temperature weather, residents of these regions are more inclined to use social media to complain, share, or seek coping strategies. In contrast, Qinghai, Tibet, and Ningxia are sparsely populated regions with unique climate conditions. In particular, Tibet and Qinghai, with their plateau climates, experience relatively fewer high-temperature days during the summer. As a result, the public in these areas may not perceive or focus on high-temperature weather as intensely as people in Guangdong and Beijing. This leads to less discussion and attention on related topics. Additionally, the lower levels of economic development, social activity, and internet usage in Qinghai, Tibet, and Ningxia contribute to the reduced discussion and attention on high-temperature-related topics Fig 2.

### 3.3. Analysis of heat attention and perception

#### 3.3.1. Spatial distribution of heat attention index.

Heat attention index and daily maximum temperature of each province have a similar spatial distribution. Guangdong Province, which experiences the highest daily temperatures, has the highest level of heat attention index. Other provinces with high average maximum temperatures, such as Hunan, Hubei, Henan, Jiangsu, Zhejiang, and Beijing, also show relatively high levels of heat attention index. Conversely, Qinghai, Tibet, Heilongjiang, Gansu, and Yunnan, with the lowest average maximum temperature, exhibit the lowest levels of heat attention index.

Sichuan Province shows relatively high heat attention index, while Guangxi, Hainan, and Taiwan have lower levels of heat attention index, displaying discrepancies compared to the temperature distribution. In Sichuan, although the overall average temperature is relatively low due to large day-night temperature difference and uneven temperature distribution within the province. High humidity in Sichuan impedes sweat evaporation, increasing the perceived heat, and strong sunlight further contributes to the discomfort. As a result, despite the overall lower average temperature, a higher volume of microblog posts related to high-temperature weather is generated in Sichuan, leading to higher heat attention index.

**Fig 3:**
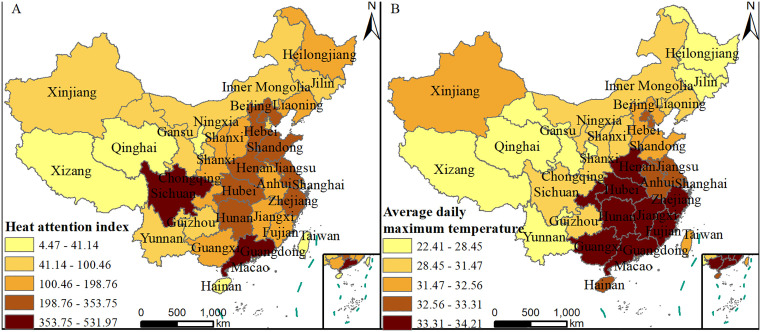
Spatial distribution of (A) heat attention index and (B) average daily maximum temperature (This figure was created using map data from Natural Earth, available at http://www.naturalearthdata.com/).

In contrast, Guangxi and Hainan, as neighboring provinces of Guangdong, experience high average temperatures but have relatively lower levels of heat attention index. This is partly due to Guangxi's lower economic development level, which may contribute to a digital divide and lower social media usage rates. Additionally, Guangxi and Hainan have lower population densities and less urbanization compared to Guangdong. Some areas may be rural with dispersed populations, leading to fewer people affected by high temperatures and consequently fewer related microblog posts, resulting in lower attention index. Taiwan, with a relatively small area and population and low microblog usage rates, has very few related posts and low levels of heat attention index Fig 3.

Heat Attention Index deliberately employs GDP-per-capita as a denominator to normalize economic disparities. This metric inherently functions as a multidimensional proxy for interrelated regional characteristics—such as urbanization rates, education levels, and digital infrastructure—which collectively shape microblog engagement. Regions with higher GDP-per-capita typically demonstrate greater urban development, advanced educational attainment, and superior broadband coverage. These factors collectively elevate social media participation. By consolidating covarying socioeconomic factors into a single proxy, this approach intentionally avoids model overcomplication while preserving computational parsimony. However, this approach has a built-in limitation: by design, the index doesn't fully capture populations with limited digital access or ability to engage online—especially vulnerable groups like young children and elderly non-social-media users, who are often highly affected by heat but lack digital channels to express it.

#### 3.3.2. Spatial differentiation of public heat tolerance.

**Fig 4:**
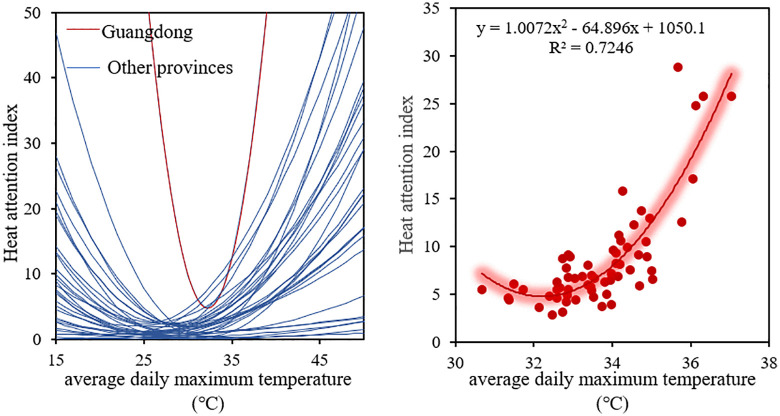
Regression model between heat attention index and average daily maximum temperature.

Using actual observed data on heat attention index and daily maximum temperatures from 34 provincial-level administrative regions in China, a quadratic function model was constructed to depict the public's response to high temperature. The study found that most regions in China exhibit similar regression trends. However, in Guangdong, the daily maximum temperature corresponding to the lowest value of heat attention index in the quadratic function model is higher than that in other provinces. As shown in Fig 4, when the daily maximum temperature is below 32.5°C, the heat attention index in Guangdong decreases with the rise in daily maximum temperature, but increases when the daily maximum temperature exceeds 32.5°C. The consistently high temperatures in Guangdong have led to a higher temperature threshold compared to other regions.

**Fig 5:**
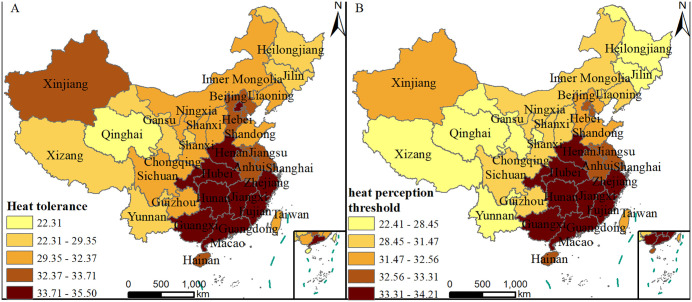
Spatial distribution of (A) Heat tolerance and (B) Heat perception threshold for 34 provincial-level administrative regions (This figure was created using map data from Natural Earth, available at http://www.naturalearthdata.com/).

To study the characteristics and differences in the distribution of public heat tolerance across different regions, the heat tolerance values for each provincial administrative region were calculated and their spatial distribution maps were plotted according to the quadratic function model of heat perception (Fig 5A). The daily maximum temperatures from July to August 2023 in each provincial administrative region were statistically analyzed to derive the average maximum temperature when the public posted hot weather-related blog posts, i.e., the public heat perception threshold (Fig 5B). Overall, the distribution patterns of heat tolerance and heat perception temperature threshold across the 34 provincial administrative regions are consistent. That is, regions with higher heat perception thresholds also exhibit higher heat tolerance. Generally, people who live in hot climates are more adapted to hot weather.

There are obvious regional differences in the public's ability to heat tolerance. Regions with higher heat tolerance are mainly distributed in the southeastern part of China, including the central regions of Henan, Hunan, and Hubei, the eastern regions of Zhejiang, Jiangxi, and Fujian, and the southern regions of Guangdong, Guangxi, Hainan, and the Macao. Chongqing and Beijing also have high heat tolerance. Notably, residents of Macao exhibit the highest heat tolerance, reaching 35.5°C. The hot and humid summer climate in these areas has led residents to gradually adapt to these conditions, thereby enhancing their heat tolerance. Moreover, buildings in these regions are often designed with heat prevention measures, such as well-ventilated structures and air conditioning, making indoor environments more comfortable, which contributes to higher heat tolerance. Provinces with lower heat tolerance are mainly located in northeastern, northwestern, and southwestern region of China, with a gradual decrease in heat tolerance from the southeast to the northwest. Among them, Qinghai has the lowest heat tolerance, with a threshold temperature of 22.3°C. Most of Qinghai is at a high altitude, where the climate is cool and dry. People living in such environments are more accustomed to low temperatures, resulting in lower heat tolerance. Additionally, Qinghai is located in the northeastern part of the Qinghai-Tibet Plateau, surrounded by mountains, with a unique climate influenced by the plateau monsoon, leading to significant temperature fluctuations and less frequent hot weather, which also contributes to the lower heat tolerance. Xinjiang in the northwest has a heat tolerance similar to that of central regions. Xinjiang, located inland, experiences high daytime temperature in summer with large day-night temperature difference, making local residents more adapted to high temperature.

#### 3.3.3. Spatial differentiation of public heat sensitivity.

**Fig 6:**
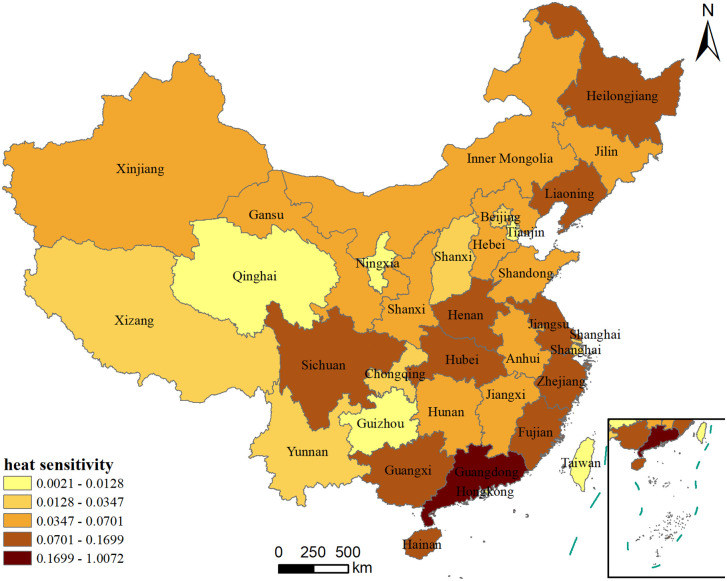
Spatial distribution of heat sensitivity in 34 provincial-level administrative regions (This figure was created using map data from Natural Earth, available at http://www.naturalearthdata.com/).

To visually display the public sensitivity to high temperatures across different regions of China, a spatial distribution map of public heat sensitivity was drawn (Fig 6). People with higher heat sensitivity are mainly concentrated in the central and eastern regions of China, such as Guangdong, Guangxi, Fujian, Sichuan, Zhejiang, and Henan provinces. Among them, the population in Guangdong Province has the highest heat sensitivity, with a sensitivity index of 1.007, followed by Guangxi (0.170), Liaoning (0.132), and Zhejiang (0.124). Coastal provinces like Guangdong, Zhejiang, and Fujian, regulated by the ocean, experience relatively moderate climate changes. However, these areas are also susceptible to climate systems such as typhoons and monsoons, which directly impact the residents' lives and safety, making them more concerned about and sensitive to temperature changes. Additionally, these regions are economically developed and densely populated, with high public expectations for quality of life. High temperatures may affect daily life, work, and health, leading to greater attention to temperature variations. Specifically, Guangdong Province, located in southern China, has a subtropical monsoon climate with hot and humid summers. The high temperature and humidity make the local people pay more attention to temperature changes, resulting in higher heat sensitivity.

People with lower heat sensitivity are primarily located in northwest of China, such as Qinghai and Ningxia provinces, as well as in southwest of China, including Chongqing and Guizhou, and in South of China, specifically Hong Kong and Macau. Among them, Hong Kong has the lowest heat sensitivity index, only 0.002, followed by Qinghai Province, with an index of 0.003. In contrast to the high heat sensitivity of Guangdong, Hong Kong and Macau have very low heat sensitivity. This discrepancy may be due to the public in these regions preferring to use other social platforms to discuss and share weather-related information, leading to limited discussion of high temperatures on Sina Microblog. This could result in a sample bias, meaning the data obtained may not fully represent the true feelings or discussion patterns of Hong Kong and Macau residents regarding high temperature. Therefore, the heat sensitivity results derived from this data may be lower than expected. Additionally, the urban infrastructure, building designs, and living habits in Hong Kong and Macau might be more conducive to coping with hot weather. For example, better air conditioning facilities, more extensive greenery, and more efficient public services may make residents feel relatively comfortable during high temperatures, leading to lower heat sensitivity. In the northwest regions, such as Tibet, Qinghai, and Ningxia, as well as the southwestern regions like Chongqing and Guizhou, the climate conditions are relatively special. Tibet and Qinghai are located on a plateau with cold and dry climates; Chongqing and Guizhou, although situated in the south, have mountainous terrain and high humidity. While these areas experience hot summers, the high humidity might make the heat less perceptible than in dry heat regions. These unique climatic conditions may make the local people have relatively low sensitivity to high temperature. People living in these areas may have developed a high tolerance to local climatic conditions due to long-term living habits and adaptation. For instance, residents of plateau regions might have a higher adaptation capacity to extreme climates due to long-term exposure to low-oxygen environments. Similarly, residents of Chongqing and Guizhou may have developed a higher tolerance to heat due to long-term living in humid environments.

## 4. Conclusion

In recent years, extreme heat events have occurred frequently, bringing serious impacts on people‘s production, life and health. Mining public heat perception characteristics from social media big data is crucial for improving government capabilities in early warning, adaptation, and management of extreme heat events. By utilizing web scraping technology to collect Sina microblog big data and applying the LDA topic model and heat perception function model, this study extracts heat-related topics, obtains indicators of heat attention, heat tolerance, and heat sensitivity, and employs geographic spatial statistical methods to reveal the spatial pattern characteristics of public heat perception across China’s provincial-level administrative regions. High temperature in Summer brings public discomfort and negative emotions. Due to climatic differences across regions, there are variations in heat tolerance and sensitivity. It is crucial to understand these regional perception differences and take timely measures to prevent heat-related health issues.

Despite its insights, this study acknowledges certain limitations that also present opportunities for future research. Primarily, the reliance on Sina Microblog data, while offering unprecedented spatial and temporal granularity, may not be fully representative of the entire population, as user demographics tend to skew towards younger, urban, and digitally active groups. Consequently, the expressed perceptions may underrepresent the vulnerabilities of critical cohorts such as the elderly or rural populations who are highly susceptible to heat impacts but less active online. To strengthen this aspect in future work, future studies could integrate multi-source data to construct a more holistic view. Specifically, promising research directions include: (1) combining social media big data with traditional social survey methods to calibrate and validate the online representation against offline realities; (2) incorporating remote sensing data (e.g., land surface temperature) to directly assess physical exposure in areas with low social media activity; and (3) leveraging fine-grained demographic and health statistics to weight the online sentiment, thereby creating a more robust and representative heat perception vulnerability index. Such a multi-method approach would be essential for developing targeted public health interventions that protect the entire population, not just the digitally vocal segment.

Notwithstanding the strategic focus on the peak summer period (July-August) for capturing the most severe heat perceptions, the temporal scope of this analysis is necessarily bounded. Future research is strongly recommended to expand the temporal dimension to encompass a full annual cycle or multiple years. Such an extension would enable the investigation of several critical phenomena beyond the scope of this study, including: the dynamics of heat awareness onset and decay throughout the year; the comparative public response to unseasonal early heatwaves versus expected peak-summer events; and the potential year-to-year variability in perception linked to differing heatwave intensities and durations. Longitudinal analysis on an annual scale would ultimately yield a more comprehensive model of public perception dynamics, significantly enhancing the practical application of findings for improving the precision of heat-health warning systems and public communication strategies across all seasons.
